# Enhancing Effective Scanning Techniques for Digital Impression in Neonates with Cleft Lip and/or Palate: A Laboratory Study Investigating the Impact of Different Scanners, Scanning Tip Sizes, and Strategies

**DOI:** 10.3390/children11121435

**Published:** 2024-11-26

**Authors:** Jyotsna Unnikrishnan, Mahmoud Bakr, Robert Love, Ghassan Idris

**Affiliations:** 1 School of Medicine and Dentistry, Griffith University, Gold Coast 4222, QLD, Australia; jyotsna.unnikrishnan@griffithuni.edu.au (J.U.); m.bakr@griffith.edu.au (M.B.); r.love@griffith.edu.au (R.L.); 2 Oral Health Service, Metro North Hospital and Health Service, Queensland Children’s Hospital, South Brisbane 4101, QLD, Australia

**Keywords:** intraoral scanning, neonates, cleft lip and palate, scanning tip size, scanning strategies, scanning time, scan stops, digital impressions

## Abstract

**Background/Objectives:** Digital impressions are increasingly used to manage Cleft lip and/or palate (CL/P), potentially offering advantages over traditional methods. This laboratory investigation sought to evaluate the impact of scanning tip sizes, different scanners, and scanning strategies on intraoral scanning in neonates with CL/P. **Methods:** Ten soft acrylic models were used to simulate the oral anatomy of neonates with CL/P, evaluating parameters such as the ability of different scanning tips to capture alveolar cleft depth, scanning time, number of scan stops, and scan quality. The study utilised various scanning tips, including the Carestream normal tip, Carestream side tip, and Trios 4 scanner tip to assess the alveolar cleft depth measurements. The Trios 4, Carestream, and iTero scanners were evaluated for the time taken, number of scan stops during cleft-unobstructed scanning and cleft-obstructed scanning. The quality of all scanned images was analysed. **Results:** The findings showed comparable accuracy in capturing alveolar cleft depth with the three-scanning tip (*p* > 0.05). Scanning time and the number of scan stops did not significantly differ across the three scanners and various scanning strategies employed (*p* > 0.05). However, scanning with the cleft obstructed required less time and resulted in fewer scan stops compared to cleft -unobstructed scanning. Despite these results, all scanners failed to record the deepest part of the alveolar cleft, highlighting a limitation in current scanning technology for neonates with CL/P. **Conclusions:** The study recommends enhancing intraoral scanning in this population by adjusting tip size, improving clinician training, optimizing protocols, and conducting further research to improve techniques.

## 1. Introduction

Cleft lip and/or palate (CL/P) is among the most prevalent craniofacial defects present in around 1 in 700 live births globally [1]. This condition can have significant implications for the affected child, necessitating comprehensive evaluation and multidisciplinary care to address its functional and aesthetic concerns [2]. Early orthopaedic management of the maxilla is essential in rehabilitating patients with CL/P, as it helps align the maxillary segments before surgical repair facilitating better surgical outcomes [3]. High-quality dental impressions are essential in this process, as they provide precise models of the child’s oral anatomy, enabling accurate assessment and treatment planning [3]. The precise evaluation of the alveolar morphology is crucial for treatment planning, especially for surgical interventions to restore normal oral function and facial aesthetics. The ability to precisely measure and evaluate these anatomical features is important for the success of corrective procedures and long-term outcomes for patients [3].

In recent years, intraoral scanning has emerged as a valuable tool in the management of CL/P, providing detailed anatomical information that facilitates treatment planning and enhances patient outcomes [4,5,6,7,8,9,10,11,12,13]. This technology offers numerous benefits compared to traditional impression methods, such as the absence of respiratory issues, enhanced patient comfort, shorter chair time, and the capability to digitally manipulate and analyse scanned data [9,10]. A recent systematic review evaluating clinician- and patient-reported outcomes indicated the comparable accuracy of digital to conventional impressions, however the ability to obtain precise intraoral scans in newborn’s with CL/P presented unique challenges [14]. Clinicians have reported that capturing the deepest portion of the alveolar cleft can be difficult, often necessitating smaller scanning tips to improve access and accuracy [15,16,17,18,19,20]. Additionally, the lack of continuity in the dental arches, recurrent motion of the child’s head, and increased salivation further complicate the scanning process in this population [16,17,18,21].

Recent studies indicate that while digital impressions in infants with CL/P achieve accuracy comparable to conventional impressions (CI), operators face significant challenges [17,20,22,23,24]. These include difficulties in recording deep cleft areas, requiring small scanning tips, and repeated attempts due to infant movement. For example, a 2020 case report on nasoalveolar moulding (NAM) in an 8-week-old with unilateral CL/P required 20 min of scanning due to these difficulties [18]. Weise et al. highlighted that CL/P patients needed longer scanning times when compared to other craniofacial anomalies and additional tools like cotton swabs to bridge cleft gaps to make the arches continuous. Additionally, achieving comprehensive scans in patients with CL/P is challenging due to small oral cavity size, frequent head movement, increased salivation, and the presence of cleft [15,17,25,26]. These challenges highlight the importance of developing ways to improve scanning efficiency and effectiveness.

The effect of varying scanning tip sizes on the ability to capture the deepest portion of the alveolar cleft remains unexplored. Additionally, the impact of different scanners and scanning strategies on scanning speed and the number of scan stops in both cleft-unobstructed and cleft-obstructed scanning has not yet been investigated.The first objective of this study were to evaluate the impact of scanning tip size has in capturing alveolar depth in newborns with CL/P. The second objective iss to evaluate the time required for scanning, the number of scan stop points during the scanning process using different scanners, and to investigate the effectiveness of various scanning strategies in capturing the areas of interest in soft acrylic models of neonates with CL/Punder two conditions; in cleft -unobstructed scanning and cleft obstructed scanning. The null hypothesis posits that different scanning tips do not impact the measurement of alveolar cleft depth and that scanning time and scan stops do not differ when using different scanners and strategies for cleft- unobstructedl and cleft-obstructed scanning.

## 2. Materials and Methods

Ethical approval was obtained from the Queensland Children’s Hospital Human Research Ethics Committee and the ethics committee at Griffith University, Queensland, Australia [HREC/24/QCHQ/108450; 4 June 2024]. All procedures complied with the ethical norms established in the Declaration of Helsinki.

The inclusion criteria comprised of models generated from babies with cleft lip and/or cleft palate (CL/P), irrespective of gender, featuring unilateral, bilateral, or midline abnormalities and varying degrees of severity. The models represented newborns who required impressions for nasoalveolar moulding (NAM) or feeding plates as part of their early cleft management at Queensland Children’s Hospital. The selection of patients was based on the requirement for NAM or feeding plates due to their cleft condition, with a range of cleft conditions.

### Cleft Lip and Palate Models

**Figure 1:**
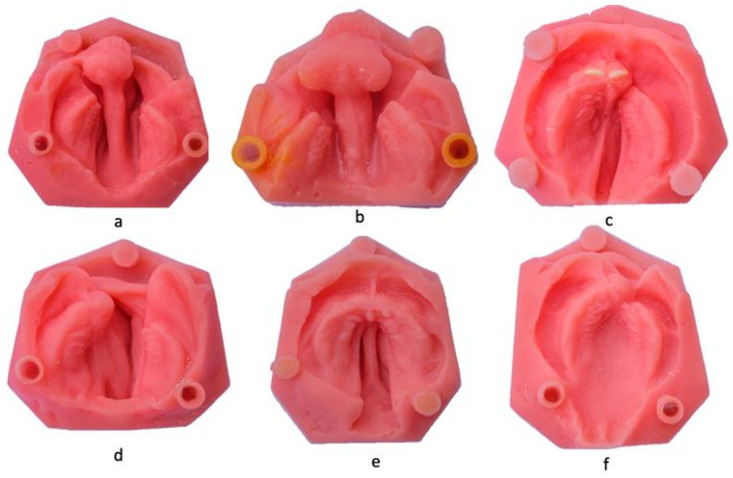
Soft Acrylic models of CL/Ps of varying severity and types: (**a**,**b**) bilateral complete cleft lip and palate; (**c**,**d**) unilateral complete cleft lip and palate; (**e**) cleft palate only; and (**f**) cleft of the alveolar ridge. (Produced at the Queensland Children’s Hospital, Children’s Oral Health Service, Metro North Hospital and Health Service).

Soft acrylic models were duplicated from original models of newborns with cleft lip and/or palate which accurately replicated the anatomical characteristics of the alveolar region facilitating controlled analysis.. The palatal, alveolar pad, and lip regions of the soft acrylic models were fabricated using soft acrylic material (Vertex Dental by 3D Systems, Soesterberg, The Netherlands) for the to simulate the flexibility and softness of oral tissues. While the model base was fabricated from rigid acrylic material (Vertex Rapid, Vertex Dental by 3D Systems, Soesterberg, The Netherlands). Figure 1.

Experiment 1: Effect of scanning tip variation on alveolar cleft depth measurement

The accuracy of various scanning tip sizes on capturing alveolar depth was assessed on models of bilateral complete cleft lip and palate (*n* = 4), unilateral complete cleft lip and palate (*n* = 4), and isolated cleft palate (*n* = 2). Three different scanner tips were utilised: Carestream regular tip (CS 3600 Carestream Dental, Atlanta, GA, USA; field of view 13 × 13 mm), the Carestream side tip (CS 3600 Carestream Dental; field of view of 13 × 7 mm), and the Trios 4 scanner tip (3Shape Dental Systems, Copenhagen, Denmark; dimensions 4.9 × 4.0 × 27.8 cm). A 3Shape E3 lab scanner (3Shape Dental Systems, Copenhagen, Denmark) was used to create a reference standard template for each model against which the scan tips were compared. All scans were saved as STL files.

A systematic scanning technique was carefully devised to ensure uniformity and comprehensiveness in scanning. To ensure documentation of both posterior ends of the arch the scan began in the tuberosity area with the greater segment of the cleft, then shifted to the opposite tuberosity (lesser segment). Scanning extended to the palatal surface of the alveolar cleft, the anterior section of the alveolar arch, and the palatal surface of the alveolar arch on the opposite side. Scanning was completed when the deepest extent of the cleft, from the posterior to the anterior region, was recorded and when the investigators satisfied with the scan quality.

**Figure 2:**
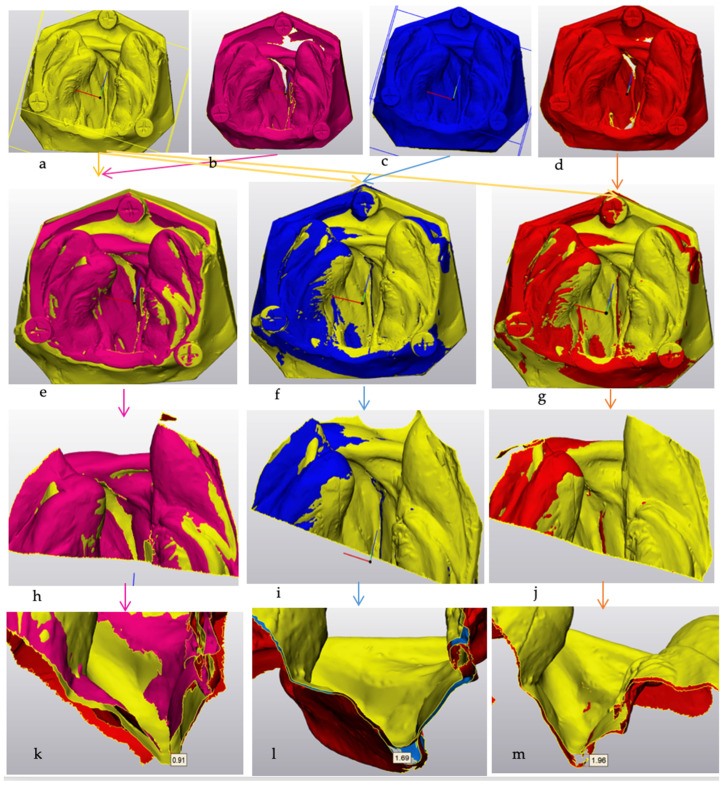
Representative scans of CL/+P models with (**a**) reference standard, (**b**) Trios4, (**c**) Carestream normal tip and (**d**) Carestream side tip. Superimposition analysis of (**e**) Trios 4 scans, (**f**) Carestream regular tip scans, (**g**) Carestream side tip scans. Cross-sectioning of superimposed image at the inter-canine line (**h**,**i**,**j**). Representative measurement of depth of the alveolar cleft (**i**) Trios 4, (**j**) Carestream regular tip, (**k**,**l**,**m**) Carestream side tip.

To compare the digital models acquired from various scanners each was superimposed over the reference standard using Materialise 3-Matic software version 16.0 (Materialise, Technologielaan, Leuven, Belgium). The superimposed images were cross-sectioned at the inter-canine line to assess the precision and depth (mm) of detail of the deepest area of the alveolar cleftusing the software measuring tools. To eliminate subjective bias, each model was measured three times, and the average of these measurements was used for statistical analysis. The measurements were repeated after two weeks for intra-examiner reliability analysis. (Figure 2).

An ANOVA test was carried out to assess the differences in cleft depth among the three scanning tips. The intra-class correlation coefficient was used to evaluate intra-examiner variability. Data analysis was performed via IBM SPSS version 29.0 with a significance level at *p* < 0.05.

Different scanners and Scanning strategy

**Figure 3:**
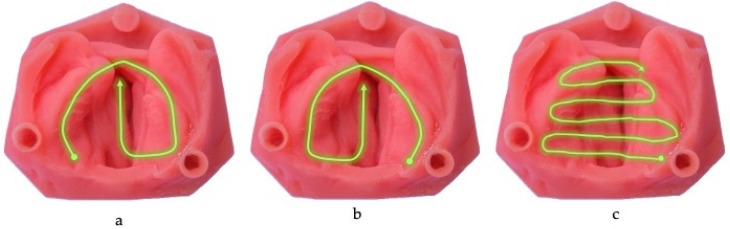
(**a**): First scanning strategy (FSS), (**b**): Second scanning strategy (SSS), (**c**): Third scanning strategy (TSS).

Three scanners were used with three different scanning strategies to capture soft acrylic models under two distinct conditions. The first condition involved scanning an unaltered model, referred to as “cleft unobstructed scanning,” where the cleft was left open. The second condition, “cleft obstructed scanning,” involved bridging the gap in the alveolar cleft by blocking the area to create a continuous arch. This obstruction was achieved either by placing a cotton roll or using a fingertip, depending on the width of the cleft defect. Three intraoral scanners were utilised: Trios 4 Trios 3 Shape (3Shape Dental Systems, Copenhagen, Denmark), iTero 5D Flex (Align Technologies, San Jose, CA, USA), and Carestream CS 3600 (Carestream Dental, Atlanta, GA, USA). Three scanning strategies were implemented: First Scanning Strategy (FSS) a predetermined protocol starting on the greater segment and continuing to the lesser segment (Figure 3a), Second Scanning Strategy (SSS) beginning on the lesser segment and continuing to the greater segment (Figure 3b), and a third scanning strategy (TSS) a modified protocol focusing on detailed scanning of the cleft area and adjacent structures (Figure 3c)

The control model and each CL/+P model was articulated in a manikin head and positioned in a dental chair to imitate the supine posture of newborns and scanned by each of the three scanners using the three different scanning strategies. The time taken for each scan and the number of scan stop points were recorded. Following this, the anterior alveolar cleft on the CL/+P models was temporarily obstructed with a fingertip or cotton roll to ensure continuity of the arches (cleft-obstructed scan) and rescanned as before. Data collected included the scanning strategy used, time taken, number of scan stop points, and the scan quality was assessed. To ensure an accurate and unbiased evaluation of the quality of images, one research team member classified the images into three categories and a second team member independently reviewed the images and verified the categorisation. The category criteria were based on if any voids or areas of poor image quality affected the image quality and completeness in capturing the anatomical areas of the cleft lip and/or palate especially the deepest part of the cleft and.

The classifications were as follows:

Category 1: SatisfactoryImages exhibited high-quality definition, capturing all areas of the CL/+P, including the deepest portion of the cleft comprehensively representing the condition and were considered ideal for clinical analysis.

Category 2: Sufficient. Images displayed sufficient clinical information, though they failed to record the deep part of the cleft but were still useful for clinical evaluation.

Images in this category

Category 3: Unsatisfactory. Images classified into this category exhibited voids or areas of poor image quality in the greater and lesser segments and were considered unsatisfactory for clinical analysis. due to the lack of necessary information.

A sample size calculation was conducted utilising G*Power software (V3.1.9.2). The estimation predicted a large effect size (d = 0.6) with an alpha level of 0.05 mm. The minimum sample size was 30 models, with ten models per group. Only seven soft acrylic models had a cleft in the palatal region; to create an adequate sample size, some models were selected to scan twice, resulting in a sample size of ten models per group. Models with bilateral complete cleft lip and palate, unilateral complete cleft lip and palate and isolated cleft palate were the models selected to be scanned twice due to the complexity, depth and prominence of the cleft(s)/defects.

ANOVA was used to evaluate the differences in scanning time and scan stops among the various scanners and scanning strategies. A two-way ANOVA was performed to examine the interaction effect of scanners and techniques. The paired T-test was used to compare the scanners and strategies for initial and cleft-obstructed scanning. Data analysis was performed via IBM SPSS version 29.0 with a significance level at *p* < 0.05.

## 3. Results

### 3.1. Effect of Scanning Tip Variation on Alveolar Cleft Depth Measurement

The analysis of variance (ANOVA) showed no statistically significant difference in the alveolar cleft depth measurement between groups, F(2,27) = 0.05, *p* = 0.95. (Table 1).

**Figure 4:**
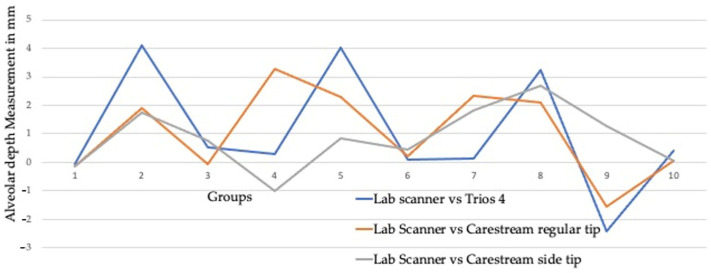
Line diagram showing the measurements across the group.

The line diagram compares alveolar depth measurements across different groups using three different scanner setups: Lab scanner vs. Carestream regular tip (red line), Lab scanner vs. Carestream small tip (green line), and Lab scanner vs. Trios 4 (blue line). Each line illustrates the variability and trends in measurements for each scanner setup across the groups in (Figure 4).

**Figure 5:**
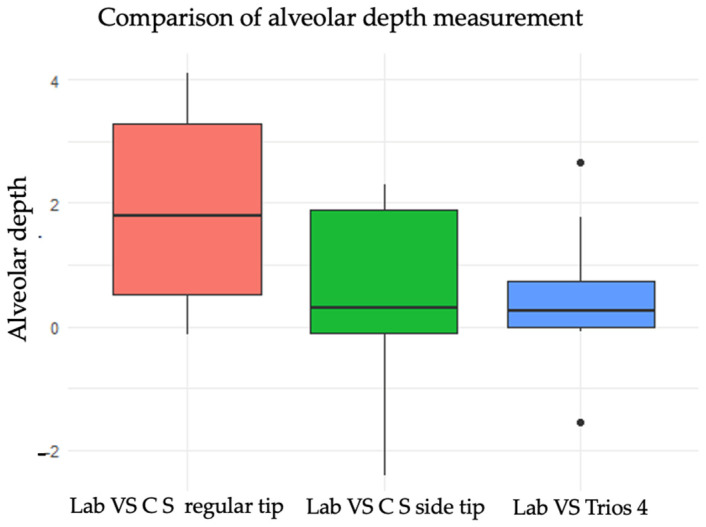
Box plot showing alveolar depth measurement with different scanning tips.

The boxplot illustrates the variability and central tendencies of alveolar depth measurements across the three different scanner setups, highlighting differences in measurement consistency and range (Figure 5). Lab vs. Carestream regular tip group shows a wider range of alveolar depth measurements, with the interquartile range (IQR) spanning from approximately 0.5 to 3.5 mm. The median depth is around 2 mm, and there are no outliers visible in this group. Lab vs. Carestream side tip group is more concentrated with the IQR ranging from around 0.5 to 2.5 mm. The median is about 1.5 mm, indicating a slightly lower central tendency than the normal tip group. There are no outliers in this group either. Lab vs. Trios 4 group has the narrowest range of measurements, with the IQR extending from about 0 to 1 mm. The median alveolar depth is close to 0.5 mm.

### 3.2. Different Scanners and Scanning Strategy

The average time taken for scanning was similar across the three scanners (*p* > 0.05, Table 1). The mean time was 1.16 min, Trios 4 had a mean time of 1.18 min iTero 1.15 min, and Carestream 1.16 min. The number of scan stops showed significant differences among the scanners. Trios 4 had the highest mean number of scan stops at 0.73, iTero 0.56 and Carestream 0.30. The mean number of scan stops for all scanners was 0.53 (±0.84) (Supplementary Table S2. ANOVA results shows that for time taken, there was no statistically significant difference between groups, F = 0.06, *p* = 0.094. For scanstops, however, there was a statitistically significant difference between groups, F = 4.12, *p* = 0.02. (Table 2).

Significant difference in scan stops was found between Trios 4 and Carestream (*p* < 0.05). No significant difference was found between Trios 4 and iTero and iTero and Carestream (*p* > 0.05 for both comparisons) (Table 3).

When comparing the strategies, the mean scanning time is very similar across the three strategies. FSS (First Scanning Strategy) has a mean of 1.17 min, SSS (Second Scanning Strategy) has a mean of 1.16 min, and TSS (Third Scanning Strategy) has a mean of 1.16 min. The overall mean time for all strategies combined is 1.16 min.

The number of scan stops also shows small differences among the strategies. FSS has a mean of 0.49 stops, SSS has a mean of 0.49 stops, and TSS has a slightly higher mean of 0.61 stops. The total mean number of combined scan stops for all strategies is 0.53.

The ANOVA results for scanning time show no significant difference between the strategies (F(2, 177) = 0.003, *p* = 0.99), indicating that the time taken for scanning does not significantly differ among FSS, SSS, and TSS. Scan Stops also show no significant difference between the strategies (F(2, 177) = 0.43, *p* = 0.65), indicating that the number of scan stops does not significantly differ among FSS, SSS, and TSS (Supplementary Table S3).

Two-way ANOVA analysis was used to analyse if there is an interaction effect between the Scanner and Strategy on the variables like time taken and number of scan stops. There are no significant main effects of Scanner (F(2, 171) = 0.05, *p* = 0.94) or Strategy (F(2, 171) = 0.003, *p* = 0.99) on time taken. Furthermore, the interaction between Scanner and Strategy is not significant (F(4, 171) = 0.03, *p* = 0.99), indicating that the effect of one variable does not depend on the level of the other variable. Similarly, there is no significant main effect of Strategy (F(2, 171) = 0.44, *p* = 0.64) on Scan Stops. Additionally, the interaction between the Scanner and Strategy is not significant (F(4, 171) = 0.56, *p* = 0.69), indicating that the effect of the Scanner does not depend on the Strategy used (Table 4).

**Figure 6:**
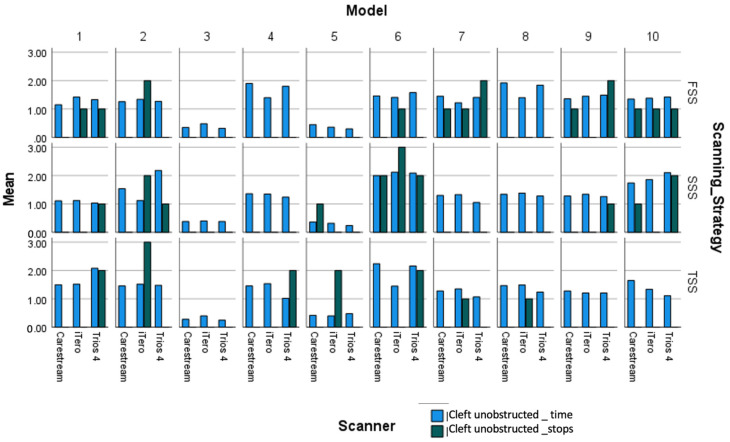
Scanning time and stops across models and strategies for cleft -unobstructed scanning.

**Figure 7:**
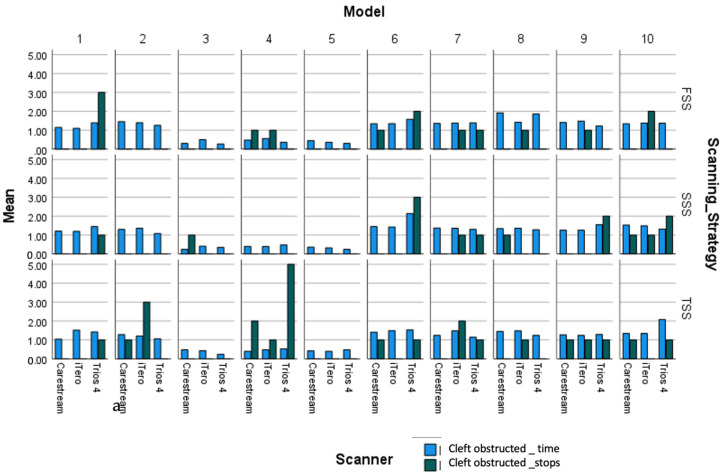
Means of scanning time(min) and mean stop points by scanners for cleft obstructed scanning.

**Figure 8:**
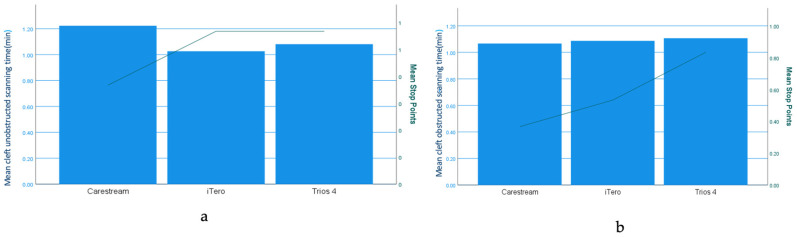
Scanning time and stops across models and strategies for cleft -unobstructed (**a**) and cleft-obstructed (**b**) scanning with three scanners.

The Dual Axes chart shows the mean scanning time and stop points for each scanner and scanning strategies (Figure 6, Figure 7 and Figure 8) and how it varies with in the ten group in cleft unobstructed scanning and cleft obstructed scanning.

All scan images were clinically sufficient, falling into category 2, as none of the images captured the deep part of the cleft defect. There were no differences observed between different scanners, scanning strategies, or whether the initial or cleft-obstructed scanning was used.

## 4. Discussion

This study explored several factors that could improve intraoral scanning in neonates with CL/P, focusing on parameters such as scanning tip size, scanning strategies, and the use of different scanners. The findings suggest that the size of the scanning tip does not significantly affect the accuracy of alveolar depth measurements. The analysis of various scanning strategies provided a comprehensive assessment of performance variables, specifically the time required for scans, the frequency of interruptions (scan stop) during the scanning process and the quality of the resulting images.

The study revealed that the scanning time remained consistent across the scanners tested and the scanning strategies employed. However, the number of scan stops varied depending on the scanner used. Notably, the Trios 4 scanner exhibited a higher frequency of scan stops compared to other devices. Notably, cleft -obstructed scanning took less time than cleft unobstructed scanning and resulted in the fewest scan stops.

Despite the different scanners and strategies, none of the scanned images successfully captured the deeper portions of the alveolar cleft. This indicates a limitation in the current scanning technology for neonates with CL/+P.

In this study, we used a lab scanner to which alveolar cleft depth measurements with different scanner tips were compared. The primary justification for using a lab scanner in this study is its role as the reference standard. Lab scanners, such as the 3Shape E3 lab scanner used in this study, are known for their high precision and accuracy in capturing detailed anatomical features [27]. Lab scanners are used to create reference models for studies, ensuring accurate comparisons with intraoral scanners [28]. They provide a comprehensive digital representation of models, assessing the performance of different tips. Lab scanners operate in a controlled environment, eliminating variables like movement, presence of saliva and lighting conditions [29]. This control ensures high-quality reference models, providing a stable basis for comparison. The lab scanner’s high detail is crucial for superimposition studies using Materialise 3-Matic software, assessing the depth and detail captured by each tip.

The Carestream regular tip, with a field of view of 13 × 13 mm, is designed to cover a broader area within the oral cavity. Its size allows for efficient scanning of larger surfaces but may present challenges when accessing narrow and deep regions, such as the alveolar cleft in neonates with CL/P. The Carestream side tip, with a reduced field of view of 13 × 7 mm, is specifically designed to access confined spaces within the oral cavity. This smaller tip size facilitates the capture of detailed anatomy in difficult-to-reach areas, making it potentially more effective in recording the deepest parts of the alveolar cleft. Despite these advantages, the study found no significant difference in the accuracy of alveolar depth measurements between the smaller and normal tips, suggesting that both tips can be used effectively, depending on the specific clinical requirements. The Trios 4 scanner tip, with dimensions of 4.9 × 4.0 × 27.8 cm, offers a balance between coverage and accessibility. Similar to the Carestream tips, the Trios 4 scanner tip demonstrated comparable accuracy in capturing alveolar depth, indicating its suitability for use in CL/P cases. However, the Trios 4 showed the closest measurements to the lab scanner among the three scanning tips. In contrast, the highest variation occurred with the Carestream regularl tip. This could be attributed to the Trios 4′s superior accuracy compared to the Carestream scanner [30]. The mechanisms by which these two scanners function vary: the Trios uses a confocal laser technique, whereas the Carestream uses triangulation techniques [31]. The impact of these mechanisms on capturing details needs to be explored further.

When evaluating the impact of the scanning tip on the accuracy of entire arch intraoral scanning, An et al. discovered that the use of a small scanner tip should be restricted to cases where it is essential [32]. A small scanner tip produces less accurate scans and necessitates a greater number of scanned images of digital impressions compared to the larger scanner tip [30,31]. A major obstacle in using intraoral scanning for newborns with CL/P is the difficulty of reaching and precisely capturing the most recessed areas of the alveolar cleft. More small scanning tips could more efficiently transverse these restricted locations. However, obtaining a thorough scan still necessitates meticulous technique and potentially many scanning endeavours. This findings in this study are important as it suggests that clinicians can use different scanning tips without compromising the quality of the digital impressions. Hence, using smaller tips may still be advantageous in accessing difficult-to-reach areas and improving overall scanning efficiency. The lack of significant differences in the measurements suggests that factors other than tip size, such as scanning strategy and operator skill, may play a more critical role in achieving high quality digital impressions in neonates with CL/P.

The accuracy of digital impressions in neonates and children with CL/P has been previously studied. Digital impressions are comparable to conventional impressions and could be a potential alternative due to reduced respiratory complications in newborns and increased acceptance by parents [10,11,17,20,22,23,24]. However, difficulties obtaining digital impressions have been reported in infants with wide clefts in cases of CL/P. [16]. Therefore, evaluating factors that could enhance the scanning process in neonates is essential. To our knowledge, this is the first study that assesses different scanning strategies and various scanners in neonates with CL/P

The First Scanning Strategy (FSS), starting on the greater segment and continuing to the lesser segment, offers a systematic approach, potentially stabilising the scanner but risking insufficient detail in the cleft area. The Second Scanning Strategy (SSS), beginning with the lesser segment and moving to the greater segment, prioritises capturing complex structures first, which may enhance initial alignment but could challenge scanner stabilisation. The Third Scanning Strategy (TSS) focuses on detailed scanning of the cleft area and adjacent structures, ensuring high accuracy where it is most critical but possibly more time-consuming. The scanning process could be affected if the alveolar cleft is deep. The scanning time with these three strategies depicted in the bar chart, even if it varies during initial and cleft-obstructed scanning, ranges from 0.3 to 2.1 min. This variation in the scanning time is due to the variation of the degree of severity of alveolar cleft between the tested models. From this observation, it is very evident that alveolar cleft in neonates with cleft lip and palate makes the scanning process challenging from the intraoral complete arch scanning in children and adults. Bridging the gap between clefts with a fingertip or cotton roll could be an effective way of scanning in this vulnerable population.

The manufactures provide instructions for the scanning sequence, which will be determined by the scanning technology of the scanners. However, certain circumstances can provide challenges in adhering to these standards, such as locations with complex contours due to severe undercuts or prioritising scanning more pleasant areas for patients before addressing the most uncomfortable parts. It was observed that using scanning techniques that differed from those specified by the manufacturer resulted in significantly reduced accuracy [33,34]. The scanning methodology varied across the Trios, Carestream, and iTero scanners. The clinical condition in neonates is peculiar due to the absence of clear landmarks, soft tissue, and the existence of deep alveolar cleft. In clinical situations, neonates often move during scanning, and increased salivation can obscure the scanning area, complicating the process further [15,35]. Thus, the choice of strategy should be tailored to the case’s specific needs, balancing accuracy, feasibility, and efficiency, with a possible combination of strategies to optimise outcomes.

The assessment of scan quality using Trios 4, iTero, and Carestream intraoral scanners in capturing cleft lip and palate (CL/P) anatomy revealed that all scans fell into Category 2, indicating clinically sufficient images that failed to capture the deepest part of the cleft defect. This consistent categorisation across all three scanners suggests a limitation in their ability to capture complex CL/P anatomy fully. With its high-speed capture and colour scanning capabilities, Trios 4 uses advanced imaging sensors that provide detailed scans in real time [36,37]. The high-speed image capture of this technology allows for more accurate data collection. However, it may face difficulties in capturing images in deeper sections of the cleft due to restrictions in light penetration and sensor reach [38]. iTero scanners employ parallel confocal imaging, a technique that collects images simultaneously at multiple depths. This technology has the potential to provide superior performance in capturing the shape and depth of the cleft. However, the results of the study indicate that even with this advanced imaging, it may not be adequate for the deepest parts of the cleft [38]. Due to the confocal concept used by both the iTero and Trios scanners, they are unable to capture clear images when the scanner tip is positioned far away from the target [37]. Carestream scanners often feature true-colour scanning with high-resolution image capture [36,39]. While this provides excellent detail and accuracy in general scanning, the limitations in capturing deeper areas of the cleft might arise from the scanner’s inability to maintain focus or light penetration in complex anatomical regions.

Intraoral scanning of edentulous ridges can pose challenges due to the absence of discernible anatomical features and the presence of smooth and level surfaces [40,41,42]. Scanning techniques to improve the edentulous arch scanning has been recommended, especially with wide palate [43,44]. The scanning techniques in neonates with CL/P can be further complicated by the lack of continuity of the edentulous arches by the cleft. Moreover, it is difficult to record the mobile soft tissues in the functional states [45,46]. In bilateral CL/P, the freely mobile premaxilla could add challenge in registering in the scan with a discontinuity of arches on both sides. Additionally, the scan quality could be affected by the light source, imaging technique, software version and image output format [47,48]. Therefore, considering these factors, proficiency in operating the scanning equipment is vital for precise scanning of neonates with CL/P, as it is necessary for effective diagnosis and planning of treatment. Research has demonstrated that the level of expertise of the operator has a substantial influence on the precision of scans produced by various intraoral scanning systems (IOSs) [49,50,51]. Furthermore, scan stops, which may arise from rapid scanning, are a prevalent problem that might impact the quality of digital impressions. Proficient operators can adjust their scanning speed to prevent these pauses, resulting in a more seamless and precise scan. The operator’s level of expertise is directly related to their ability to adjust scanning speed and technique. When scanning newborns with CL/P, these findings emphasise the significance of the operator’s expertise in creating precise and dependable digital impressions. Due to the intricate nature of neonatal anatomy and the intricate nature of CL/P cases, the presence of a skilled operator can significantly improve the quality of the scans, resulting in improved treatment outcomes.

Based on this study’s findings, several recommendations could be included in clinical scenarios to enhance intraoral scanning in neonates with CL/P. First, tailoring the tip size to the clinical needs is crucial, as the study indicates no significant difference in measurement accuracy among the different tips. Selecting the appropriate tip size based on specific anatomical challenges and clinical goals is essential. For instance, using smaller tips for detailed scans of deep cleft areas and normal tips for broader surface areas can improve the overall scanning process. Second, enhanced training for clinicians on the use of different scanning tips and techniques is vital irrespective of the type of scanners. This includes familiarising clinicians with the unique challenges of scanning neonates and developing skills to manage movement and salivation effectively. Operator proficiency is directly related to scanning accuracy, and proficient operators can adjust their scanning speed to avoid scan stops, resulting in more seamless and precise scans. Third, optimising scanning protocols by developing standardised procedures that incorporate the use of various tip sizes, specific strategies for different regions of the oral cavity, and bridging the gap between cleft with fingertip or cotton roll can improve scanning efficiency. Lastly, future research should focus on clinical studies refining scanning techniques, exploring new tip designs, and evaluating the long-term impact of different scanning methods on treatment outcomes in CL/P patients. These recommendations, derived from the study findings, aim to improve the quality of digital impressions and enhance the diagnosis and treatment planning for neonates with CL/P.

This study is the first known investigation to examine the impact of various scanning tip sizes, scanners, and techniques on intraoral scanning in newborns with cleft lip and palate (CL/P). Prior research has mostly concentrated on evaluating the accuracy of digital imprint technology. Furthermore, the existing literature does not provide any information regarding the elements that could enhance the efficacy of scanning in infants with CL/P. This study is significant because it is the first extensive analysis of factors that impact intraoral scanning devices, primarily in the context of cleft lip and palate cases. Our research addresses a significant gap in the current literature by thoroughly assessing the factors that influence the ability of intraoral scanners to accurately capture the complex and difficult anatomy associated with CL/P. The findings of this study highlight the existing technology constraints and have the potential to provide valuable insights for both clinical practices and the advancement of next-generation scanning technologies that effectively tackle the distinct issues presented by CL/P.

Our study’s findings offer valuable insights despite some inherent limitations. The results of this study are restricted by its in vitro character, which may not accurately reflect the clinical validity of intraoral scanning in new-borns with CL/P. This study utilised soft acrylic models to accurately replicate the oral conditions found in newborns with cleft lip and palate CL/P. Our objective was to improve the accuracy of our findings by employing soft acrylic models that may simulate real-world clinical circumstances, therefore minimising the differences between experimental conditions and actual practice. Although these models offer a controlled experimental environment, they may not completely reproduce the intricacy and diversity of the oral anatomy in real patients. Additionally, mounting these soft acrylic models in a manikin head while testing scanning strategies with different scanners helps to simulate the clinical conditions of neonates with CL/P. The manikin was kept in a supine position to better mimic the actual patient scenario. Clinicians should be aware of the challenges associated with scanning neonates with CL/P and strive to improve scanning efficiency and accuracy through proper training and technique refinement. Conducting in vitro research can be beneficial in clinical circumstances since they provide a controlled environment and minimise the compounding factors. [52] However, to validate the findings of this study and improve scanning techniques in newborns with CLP, it is necessary to do additional research using bigger sample sizes and clinical data from real patients.

## 5. Conclusions

The study highlights that different scanning tips, including the Carestream normal tip, Carestream side tip, and Trios 4 scanner tip, exhibit comparable accuracy in capturing alveolar depth in neonates with cleft lip and palate (CL/P).Smaller scanning tip is recommended in neonates with cleft lip and palate.

Additionally, the study found that the time taken and the number of scan stops did not differ significantly across the three scanners and the various scanning strategies employed. All scanners were able to capture clinically sufficient scan quality; however, less time and fewer scan stops were required in cleft-obstructed scanning. Nonetheless, all scanners failed to document the deepest part of the cleft.

Future studies should develop intraoral scanners for capturing intricate details of cleft anomalies in neonates, considering unique challenges like small mouth size, frequent movements, increased salivation, and wide and deep clefts.

## Figures and Tables

**Table 1:** ANOVA statistical results for comparing the cleft depth measurement between groups, including the Sum of Squares, degree of freedom (df), mean square (measured in mm), F-values, and significance levels (Sig).

	Sum of Squares	df	Mean Square	F	Sig.
Between Groups	0.26	2	0.128	0.05	0.95
Within Groups	70.93	27	2.63		
Total	71.18	29			

**Table 2:** ANOVA results comparing mean differences for the time taken and the scan stops among different intraoral scanners, including the Sum of Squares, degree of freedom (df), mean square (measured in mm), F-values and significance levels (Sig).

		Sum of Squares	df	Mean Square	F	Sig.
Time_Taken	Between Groups	0.030	2	0.01	0.06	0.94
	Within Groups	46.52	177	0.26		
	Total	46.54	179			
Scan_stops	Between Groups	5.733	2	2.86	4.123	0.02
	Within Groups	123.06	177	0.69		
	Total	128.80	179			

**Table 3:** Tukey HSD multiple comparisons of mean differences for the time taken and scan stops between different intraoral scanners: Trios 4, iTero, and Carestream.

Dependent Variable	(I) Scanner	(J) Scanner	Mean Difference (I-J)	Std. Error	Sig.	95% Confidence Interval	
						Lower Bound	Upper Bound
Time_Taken	Trios 4	iTero	0.032	0.093	0.939	−0.18	0.25
		Carestream	0.014	0.093	0.988	−0.20	0.23
	iTero	Trios 4	−0.032	0.093	0.939	−0.25	0.19
		Carestream	−0.02	0.093	0.980	−0.23	0.20
	Carestream	Trios 4	−0.014	0.093	0.988	−0.23	0.21
		iTero	0.02	0.093	0.980	−0.20	0.24
Scan_stops	Trios 4	iTero	0.17	0.15	0.519	−0.19	0.52
		Carestream	0.43	0.15	0.014	0.07	0.79
	iTero	Trios 4	−0.16	0.15	0.519	−0.52	0.19
		Carestream	0.26	0.15	0.189	−0.09	0.63
	Carestream	Trios 4	−0.43	0.15	0.014	−0.79	−0.07
		iTero	−0.26	0.15	0.189	−0.63	0.09

**Table 4:** Two-way ANOVA tests of between-subjects effects for the time taken, evaluating the impact of different scanners, strategies, and their interaction.

**Dependent Variable: Time_Taken**					
**Source**	**Type III Sum of Squares**	**df**	**Mean Square**	**F**	**Sig.**
Corrected Model	0.070 ^a^	8	0.009	0.032	1.000
Intercept	244.961	1	244.96	901.26	0.000
Scanner	0.030	2	0.015	0.056	0.946
Strategy	0.002	2	0.001	0.003	0.997
Scanner Strategy	0.038	4	0.009	0.035	0.998
Error	46.477	171	0.272		
Total	291.570	180			
Corrected Total	46.547	179			
a. R Squared = 0.002 (Adjusted R Squared = −0.045)					
**Dependent Variable: Scan_Stops**					
**Source**	**Type III Sum of Squares**	**df**	**Mean Square**	**F**	**Sig.**
Corrected Model	7.949 ^a^	8	0.994	1.406	0.197
Intercept	51.097	1	51.097	72.300	0.000
Scanner	5.761	2	2.880	4.076	0.019
Strategy	0.632	2	0.316	0.447	0.640
Scanner Strategy	1.589	4	0.397	0.562	0.691
Error	120.851	171	0.707		
Total	180.000	180			
Corrected Total	128.800	179			
a. R Squared = 0.062 (Adjusted R Squared = 0.018)					

## Data Availability

The work includes all the original contributions made, and any additional questions can be forwarded to the corresponding author.

## Supplementary Materials

The following supporting information can be downloaded at: https://www.mdpi.com/article/10.3390/children11121435/s1, Table S1: Multiple comparisons of mean differences in variable values between a lab scanner, Trios 4, Carestream Normal tip, and Carestream side tip using Tukey HSD test; Table S2: Descriptive statistics for the time taken (in minutes) and the number of scan stops across three different intraoral scanners including the mean, standard deviation, standard error, 95% confidence intervals for the mean, and the range (minimum and maximum) of the observed values for each scanner; Table S3: ANOVA results comparing mean differences for scanning time and scan stops among different scanning strategies including the Sum of Squares, degree of freedom (df), mean square (measured in mm), F-values, and significance levels (Sig); Table S4: Paired *t*-test to compare the time taken with different scanners and strategies between initial scanning and cleft obstructed scanning. Table S5: Paired-sample effect sizes to quantify the magnitude of difference in time with scanners and strategies between initial scanning and cleft-obstructed scanning; Table S6: Paired *t*-test to compare the scan stops with different scanners and strategies between initial scanning and cleft- obstructed scanning; Table S7: Paired-sample effect sizes to quantify the magnitude of difference in time with scanners and strategies between initial scanning and cleft -obstructed scanning.
