# Supplementary material for: Enhancing Effective Scanning Techniques for Digital Impression in Neonates with Cleft Lip and/or Palate: A Laboratory Study Investigating the Impact of Different Scanners, Scanning Tip Sizes, and Strategies
Source: Children (Basel). 2024 Nov 26;11(12):1435. doi: 10.3390/children11121435 (PMC11674068; doi:10.3390/children11121435)
Supplement: Supplementary file 1 [file children-11-01435-s001.zip › children-3296063-supplementary.pdf]

Multiple Comparisons

Dependent Variable: Variables value

Tukey HSD

(I) Groups	(J) Groups	Mean Difference (I-J)	Std. Error	Sig.	95% Confidence Interval	
					Lower Bound	Upper Bound
Lab scanner vs Trios 4	Lab scanner vs CarestreamNormal tip	-.01270	.72484	1.000	-1.8099	1.7845
	Lab scanner vs Carestream side tip	.18900	.72484	.963	-1.6082	1.9862
Lab scanner vs Carestream Normal tip	Lab scanner vs Trios 4	.01270	.72484	1.000	-1.7845	1.8099
	Lab scanner vs Carestream sidel tip	.20170	.72484	.958	-1.5955	1.9989
Lab scanner vs Carestream side tip	Lab scanner vs Trios 4	-.18900	.72484	.963	-1.9862	1.6082
	Lab scanner vs Carestream Normaltip	-.20170	.72484	.958	-1.9989	1.5955

Supplementary Table S1*:* Multiple comparisons of mean differences in variable values between a lab scanner, Trios 4, Carestream Normal tip, and Carestream side tip using Tukey HSD test.

Supplementary table S2:

Descriptives

		N	Mean	Std. Deviation	Std. Error	95% Confidence Interval for Mean Lower Bound	Upper Bound	Minimum	Maximum
Time_Taken	Trios 4	60	1.1818	.57263	.07393	1.0339	1.3298	.24	2.18
	iTero	60	1.1502	.45461	.05869	1.0327	1.2676	.32	2.12
	Carestream	60	1.1682	.50383	.06504	1.0380	1.2983	.24	2.24
	Total	180	1.1667	.50994	.03801	1.0917	1.2417	.24	2.24
Scan_stops	Trios 4	60	.7333	1.03934	.13418	.4648	1.0018	.00	5.00
	iTero	60	.5667	.85105	.10987	.3468	.7865	.00	3.00
	Carestream	60	.3000	.53043	.06848	.1630	.4370	.00	2.00
	Total	180	.5333	.84826	.06323	.4086	.6581	.00	5.00

Supplementary table S2: Descriptive statistics for the time taken (in minutes) and the number of scan stops across three different intraoral scanners including the mean, standard deviation, standard error, 95% confidence intervals for the mean, and the range (minimum and maximum) of the observed values for each scanner

Supplementary Table S3:

ANOVA

		Sum of Squares	df	Mean Square	F	Sig.
Time_Taken	Between Groups	.002	2	.001	.003	.997
	Within Groups	46.54	177	.263		
	Total	46.54	179			
Scan_stops	Between Groups	.625	2	.313	.432	.650
	Within Groups	128.17	177	.724		
	Total	128.80	179			

Supplementary Table S3: ANOVA results comparing mean differences for scanning time and scan stops among different scanning strategies including the Sum of Squares, degree of freedom(df) mean square(measured in mm)F-values and significance levels(Sig).

Supplementary Table S4

Paired Samples Test

		Paired Differences					t	df	Sig. (2-tailed)
		Mean	Std. Deviation	Std. Error Mean	95% Confidence Interval of the Difference Lower	Upper			
Pair 1	Time Taken Scanner	--.83328	.96933	.07225	-.97585	-.69071	-11.533	179	.000
Pair 2	Time Taken Strategy	--.83883	.96432	.07188	-.98067	-.69700	-11.671	179	.000

Supplementary Table S4 :Paired T test to compare the time taken with different scanners and strategies between initial scanning and cleft obstructed scanning

Supplementary Table S5

Paired Samples Effect Sizes

			Standardiser	Point Estimate	95% Confidence Interval Lower	Upper
Pair 1	Time Taken - Scanner	Cohen's d	.96933	-.860	-1.030	-.688
		Hedges' correction	.97136	-.858	-1.028	-.686
Pair 2	Time Taken - Strategy	Cohen's d	.96432	-.870	-1.041	-.697
		Hedges' correction	.96635	-.868	-1.038	-.696

Supplementary Table S5: Paired sample effect sizes to quantify the magnitude of difference in time with scanners and strategies between initial scanning and cleft obstructed scanning

Paired Samples Test

Paired Differences	t	df

		Mean	Std. Deviation	Std. Error Mean	95% Confidence Interval of the Difference				Sig. (2-tailed)
					Lower	Upper			
Pair 1	Scan_stops Scanner	-- 1.46 667	1.29632	.09662	-1.65733	-1.27600	-15.179	179	.000
Pair 2	Scan_stops Strategy	-- 1.47 222	1.14057	.08501	-1.63998	-1.30447	-17.318	179	.000

Supplementary Table S6

Supplementary Table S7:

Supplementary Table S6: Paired T test to compare the scan stops with different scanners and strategies between initial scanning and cleft obstructed scanning.

Supplementary Table S7: Paired sample effect sizes to quantify the magnitude of difference in time with scanners and strategies between initial scanning and cleft obstructed scanning

Paired Samples Effect Sizes

			Standardiser	Point Estimate	95% Confidence Interval Lower	Upper
Pair 1	Scan stops - Scanner	Cohen's d	1.29632	-1.131	-1.318	-.943
		Hedges' correction	1.29904	-1.129	-1.315	-.941
Pair 2	Scan stops - Strategy	Cohen's d	1.14057	-1.291	-1.488	-1.092
		Hedges' correction	1.14297	-1.288	-1.485	-1.089
